# Nonfunctional ingestion of plant miRNAs in silkworm revealed by digital droplet PCR and transcriptome analysis

**DOI:** 10.1038/srep12290

**Published:** 2015-07-21

**Authors:** Ling Jia, Dayan Zhang, Zhonghuai Xiang, Ningjia He

**Affiliations:** 1 State Key Laboratory of Silkworm Genome Biology, Southwest University, Beibei, Chongqing 400715, P. R. China

## Abstract

Since a plant miRNA (miR168) cross-regulating a mammalian transcript was reported, miRNA-mediated cross-kingdom communication has become one of the most compelling but controversial topics. In the present study, we used silkworm and mulberry, which is a model for studies on the interactions between the insect and its host plant, to address whether miRNA-mediated cross-kingdom communication is a common phenomenon. The results of TA clone, Sanger sequencing and droplet digital PCR demonstrated that several mulberry-derived miRNAs could enter to silkworm hemolymph and multiple tested tissues. Synthetic miR166b was also detected in hemolymph and fat body. However, the ingestion of synthetic miR166b did not play roles in silkworm physiological progress, which was revealed by RNA-seq analyses, RT-PCR, and phenotypic investigations. Mulberry miRNAs are convincingly transferred to the silkworm orally and no physiological process associated with the miRNAs was demonstrable. The results provided a new aspect of cross-kingdom miRNA transfer.

miRNAs, which are a class of ~22 nt non-coding small RNA molecules, play vital roles by compounding to transcripts of target genes to inhibit translation or degrade mRNA of target genes in animals and plants^1^. At present, the studies of miRNAs are mainly focusing on the intracellular miRNAs functions in their own species. For example, miRNAs regulate the growth and development of root, leaf, and flower in plants^2^,^3^,^4^, and have important roles in the development of organs in animals^5^,^6^. Recently, released miRNA was reported to be secreted from cells to body fluid and circulate in body fluid in a surprisingly stable-state in mammals^7^,^8^. Those miRNAs could be carried by lipoprotein or other RNA-binding molecules^9^,^10^, or packed by microvesicles to deal with the harsh condition (e.g., RNase, and extreme pH)^10^. Circulating miRNAs in body fluid were guided to target cells to regulate the expression of target genes^9^,^11^.

Recently, miRNA-mediated cross-kingdom communication has become an interesting but controversial topic since it was first reported in 2011^12^. Zhang *et al.* discovered that rice-derived miRNAs cross the mammalian gastrointestinal tract to the mouse bloodstream, liver, and other tissues, where they regulate cholesterol levels by reducing the amount of low-density lipoprotein receptor-associated protein 1 (LDLRAP1)^12^. These findings provide new insights for genetic regulation by food ingestion and raise the prospect of engineering food or using oral small nucleic acid to prevent or cure diseases^13^,^14^. However, in several other recent studies of mice, pigtailed macaques, or insects (*Helicoverpa zea* and *Spodoptera frugiperda*), plant-derived miRNAs were not detected after ingestion of plant-derived food^13^,^15^,^16^. In this context, we used silkworm, *Bombyx mori*, an oligophagous insect that feeds only on mulberry leaves, to study whether mulberry miRNAs can transfer to silkworm upon ingestion. TA clone, Sanger sequencing and droplet digital PCR assay were used to detect mulberry miRNAs in silkworm subset of tissues. RNA-seq was then performed to explore the physiological progresses associated with one of plant miRNA, miR166b, in silkworm.

## Results

### Mulberry-derived miRNAs were detected in silkworm hemolymph

We aligned silkworm hemolymph small RNA sequences (GSE48168)^17^ with mulberry miRNAs revealed by next generation sequencing^18^. To our surprise, eight mulberry miRNAs (mno-miR166c, mno-miR166b, mno-miR167e, mno-miR396b, mno-miR159a, mno-miR162, mno-miR156c, and mno-miR398) were identified in silkworm hemlolymph (Table 1). The reads of these miRNAs were relatively low, with the highest being 11. To ensure that these mulberry-derived miRNAs do not represent contamination during solexa sequencing or sample preparation, we used stem-loop PCR to clone the eight mulberry miRNAs, generating 50 TA-clones for each miRNA, and then subjected them to Sanger sequencing. All of the miRNAs except for mno-miR162 could be detected, with positive clone ratios (perfectly matched clones/total clones) ranging from 35.90% to 70.83% (Table 2, 1^st^ sequencing Hemolymph). Positive clones with incorrect sequences could be explained by the relative low contents of these miRNAs or to homologous silkworm derived small RNAs. As control, we also cloned four mulberry miRNAs that were not identified in the silkworm hemolymph and have varied expression levels in mulberry leaves^18^. There were no positive clones for miR535, miR168b, and miR172a (Table 2, 1^st^ sequencing Hemolymph), and the positive clone ratio of miR169a was 5.13%, which is much lower than other seven miRNAs (Table 2, 1^st^ sequencing Hemolymph). These results confirm the specificity of the seven mulberry miRNAs (miR166c, miR166b, miR167e, miR396b, miR159a, miR156c, and miR398). To further confirm these results, we re-cloned miR169a, miR166b, miR166c, miR167e, and miR396b in silkworm hemolymph. The sequencing frequencies of the five miRNAs were similar to those of the first sequencing run (Table 2, 2^nd^ sequencing Hemolymph).

### Mulberry-derived miRNAs were detected in multiple silkworm tissues

As the silkworm is a non-vertebrate with an open circulatory system, its organs and tissues are suspended in the hemolymph. To explore whether plant miRNAs enter into the silkworm tissues, the seven mulberry miRNAs were cloned in the fat body and silk gland. The sequencing frequency of four miRNAs (miR166b, miR166c, miR396b, and miR167e) ranged from 12.50% to 73.17% in fat body, and from 13.51% to 72.22% in silk gland, which is significantly higher than frequency of the control, mno-miR169a (2.63% in fat body; 0.00% in silk gland) (Table 3, 1^st^ sequencing Fat body and Silk gland). Repeat sequencing verified the overall trend that mno-miR166b, mno-miR166c and mno-miR396b were more abundant than the control mno-miR169a in the fat body and silk gland, although the frequencies fluctuated in the two experiments (Table 3, 2^nd^ sequencing Fat body and Silk gland). In addition, these four mulberry miRNAs were also cloned in other seven silkworm tissues including brain, prothoracic gland, salivary gland, gut, malpighian tubule, ovary, and testis. The sequencing frequency of four miRNAs (miR166b, miR166c, miR396b, and miR167e) ranged from 25.64%–87.23% in brain, 9.76%–66.67% in prothoracic gland, 6.67%–36.84% in salivary gland, 8.11%–31.71% in gut, 10.81%–48.78% in malpighian tubule, 7.32%–34.09% in ovary, and 8.16%–37.50% in testis, which was more abundant than the control mno-miR169a (Table 3, supplementary Table S1). The results support the assertion that mulberry miRNAs can enter into silkworm tissues.

### Synthetic miR166b was detected in silkworm hemolymph and fat body

**Figure 1:**
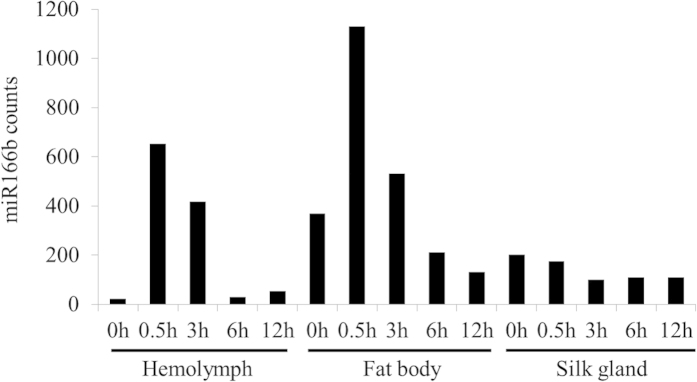
Synthetic miR166b entry into silkworm hemolymph and fat body after silkworm ingestion as determined by droplet digital PCR. The copy number counts of miR166b in 80 ng small RNA of silkworm hemolymph, 240 ng total RNA of fat body, and 240 ng total RNA of silk gland were investigated by droplet digital PCR before (0 h) and following ingestion of 300 pmol synthetic miR166b (0.5 h, 3 h, 6 h, and 12 h).

To further investigate the potential role of miR166b, we synthesized this miRNA with modified 2’-O-methyl on its terminal nucleotide, which is characteristic of plant miRNAs^19^, and smeared 300 pmol on a piece of mulberry leaf to feed silkworm larvae. Droplet digital PCR was applied to detect the copy numbers of miR166b in silkworm hemolymph, fat body, and silk gland before and after ingestion. The copy number counts differed over time according to the tissue type (Fig. 1, supplementary Fig. S1 and Table S2). In hemolymph, the miR166b counts peaked at 0.5 h and 3 h, with 30 and 18 times the count at 0 h; the counts at 6 h and 12 h were similar to those at 0 h. The trend in fat body was similar to that in hemolymph. However, the trend in silk gland differed, with miR166b counts at 0.5 h, 3 h, 6 h, and 12 h slightly less than the count at 0 h. These results suggest that synthetic miR166b could enter to silkworm hemolymph and fat body, but not to silk gland. These results also suggest that it takes 0.5 h or less for synthetic miR166b to enter hemolymph and fat body and that miR166b lasts 3 hours before degradation.

### Expression of miR166b potential target genes were not repressed by the synthetic miR166b

**Figure 2:**
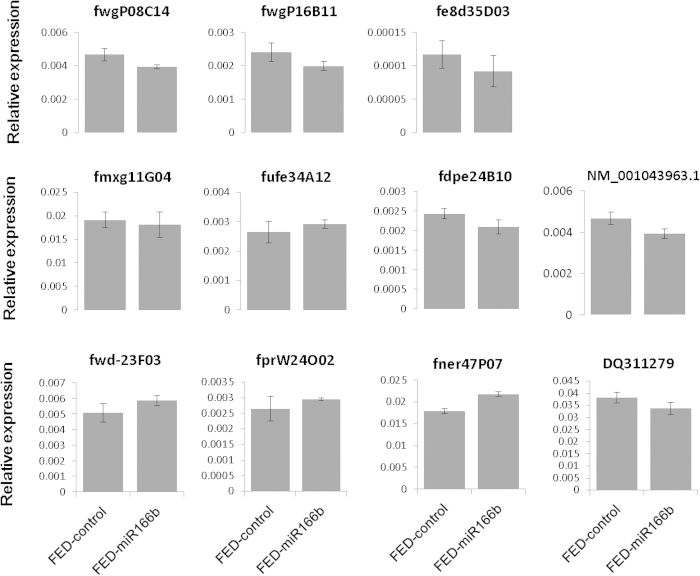
The expression level of 11 potential miR166b target genes in whole body before and after silkworm ingested synthetic miR166b. FED-control represents the silkworm larvae were fed on only mulberry leaves. FED-miR166b means the silkworm larvae were fed on a piece of mulberry leaf containing synthetic miR166b. Statistical significance was determined by Student’s t test (*p < 0.05).

To explore the functions of miR166b in silkworm, we predicted the potential target genes for miR166b in silkworm full-length cDNA data using miranda software. With the criteria of energy lower than −25 kcal/mol and perfected “seed region” match, we identified 72 potential target genes of miR166b (data not shown). Eleven best matched target genes, whose energy were lower than −26.84 kcal/mol (supplementary Table S3) were subjected to RT-PCR verification. The results showed the expression levels of these genes were not significantly changed in silkworm fed on synthetic miR166b (Fig. 2, supplementary Fig. S2).

### RNA-seq analyses of silkworm fed on synthetic miR166b

**Figure 3:**
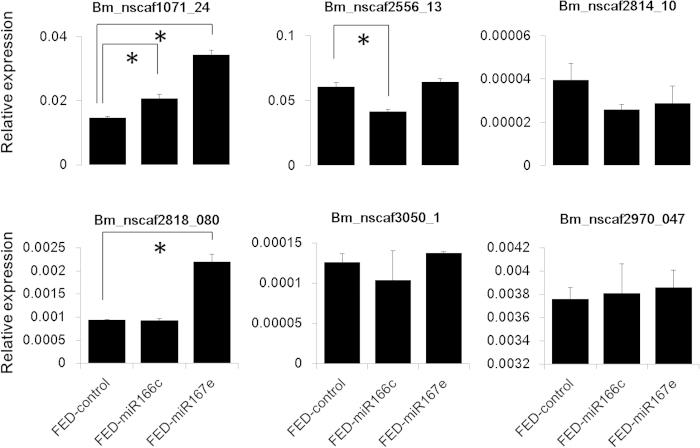
The expression level of 6 differentially expressed genes in silkworm ingested synthetic miR166c and miR167e. Differentially expressed genes were identified by transcriptome analysis. FED-control represents the silkworm larvae were fed on only mulberry leaves. FED-miR166c and FED-miR167e means the silkworm larvae were fed on a piece of mulberry leaf containing synthetic miR166c and miR167e, respectively. Statistical significance was determined by Student’s t test (*p < 0.05).

For a better understanding of the potential overall biological roles of ingested mno-miR166b in silkworms, we used RNA-seq to reveal the global expression patterns in whole body of silkworm larvae before and after feeding with synthetic miR166b for 3 hours. Compared with silkworm larvae fed on mulberry leaves, only 30 genes were differentially expressed, of which 17 were up-regulated and 13 were down-regulated in larvae feeding with synthetic miR166b. These genes can be classified into seven groups (supplementary Fig. S3, Table 4): immunity (11 genes), response to stress (1 gene), development (5 genes), cytoskeleton (1 gene), digestion (1 gene), hydrolyzing juvenile hormone (1 gene), and unknown function (10 genes). Genes involved in immunity and stress comprised about 40% of the differentially expressed genes. Thus, the ingestion of synthetic miR166b might activate the host response against non-self-molecules. We further fed silkworm larvae with two other synthetic miRNAs, miR166c and miR167e. Six genes randomly chosen from 30 DEGs (Differentially Expressed Genes) were subjected to RT-PCR analyses. The result showed that *Bm_nscaf2556_13* was down-regulated by synthetic miR166c, *Bm_nscaf2818_080* was up-regulated by miR167e, and *Bm_nscaf1071_24* gene was up-regulated by both (Fig. 3). The results indicated that the different expression of these immunity-related genes was caused by the ingestion of nucleic acids, not specific to miR166b. Therefore, we speculate that the ingested synthetic miR166b basically did not play a significant physiological role to *B. mori*. Consistently, there was no significant overlap in the predicted miR166b target sites for the 30 differentially expressed genes (data not shown). In addition, the expression levels of 72 potential target genes for miR166b were not significantly changed in silkworm fed on syntetic miR166b (supplementary table S4). These results suggest that mulberry miR166b has no significant effect on the expression of silkworm genes.

### Phenotypic investigation of silkworms fed on synthetic miR166b

**Figure 4:**
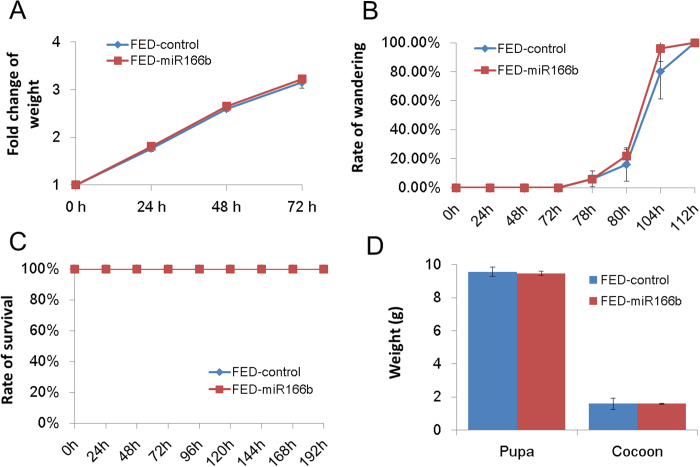
The phenotypic investigation of silkworm larvae fed on mulberry leaves with/without synthetic miR166b. The phenotypic investigation including larva weight (**A**), rate of wandering (**B**), rate of survival (**C**), pupa and cocoon weight (**D**) were measured before (0 h) and after silkworm ingested synthetic miR166b (24 h, 48 h, 72 h, 96 h, 120 h, 144 h, 168 h, and 192 h). Fold change of weight means the ratio of lavae weight after silkworm ingested synthetic miR166b (24 h, 48 h, 72 h) divided the larvae weight at 0 h. Rate of wandering indicates the percentage of silkworm developed into the wandering stage. FED-control represents the silkworm larvae were fed only on mulberry leaves. FED-miR166b means the silkworm larvae were fed on a piece of mulberry leaf containing synthetic miR166b. Statistical significance was determined by Student’s t test (*p < 0.05).

We also investigated the phenotypic changes of silkworm feeding on mulberry leaves with and without synthetic miR166b. As shown in Fig. 4, neither the weight (Fig. 4A) nor their wandering rate (Fig. 4B) showed significant difference between two groups. Similar observations had been made in lethality (Fig. 4C). The fat body and silk gland are two major organs in silkworm. The developmental growth of silk gland and fat body can be reflected by the weight of cocoon and pupae, respectively. Since the synthetic miR166b was detected in fat body and not silk gland, we then measured the pupa and cocoon weight at 10 days after ingestion of synthetic miR166b. Results showed that there was no significant difference of pupa and cocoon weight between two groups (Fig. 4D).

## Discussion

Using T-A clone and Sanger sequencing assay, we revealed that mulberry-derived miRNAs not only transferred to silkworm hemolymph, but also to other tissues such as fat body and silk gland. In addition, droplet digital PCR assay showed that synthetic miR166b also could enter to silkworm hemolymph and fat body after orally feeding. However, RNA-seq analyses, RT-PCR verification, and phenotypic investigation showed that no physiological process in silkworm associated with the ingestion of one tested mulberry miRNA (miR166b). Zhang *et al.* (2012) analyzed small RNA libraries of several species, and they concluded that low reads of plant-derived miRNA detected in animals were from contamination of large-scale sequencing and sample preparation^16^. Dickinson *et al.* (2013) applied solexa method to sequence the serum and liver of mice fed with three synthetic food containing different amount of rice^13^. They also emphasized those low reads of plant-derived miRNA in mammal tissues were from contamination^13^. However, the above two studies did not use different methods to detect the plant-derived miRNAs in animals. The methods used in this study are much more rigorous than those of previous studies and the data proved that the mulberry-derived miRNAs detected in silkworm did not result from contamination as following reasons. (1) TA-cloning and Sanger sequencing can rule out the possibility that the mulberry-derived miRNAs in silkworm were from solexa sequencing contamination; (2) Mulberry miRNAs with high expression levels were used as controls and were not detected in the silkworm hemolymph; (3) The identification of synthetic miR166b was confirmed in silkworm tissues after ingestion.

Our results demonstrated that both of mulberry-derived and synthetic plant miRNA were convincingly transferred to silkworm tissues. Zhang *et al.* and Zhou *et al.* recently reported that rice and honeysuckle-derived miRNAs could be detected in mammalian tissues^12^,^20^. Taken their results and our data together, it is undoubtedly believed that plant-derived miRNAs entering to recipient animal organisms is a conserved phenomenon. But nevertheless there are some differences between our observation and Zhang *et al.*’ report^12^. One is the plant-derived miRNAs detected in silkworm were less than those reported by Zhang *et al.* in mammal^12^. A total of seven miRNAs were identified in the silkworm hemolymph and only two plant-derived miRNA (mno-miR166b and mno-miR156c) are common in silkworm and mammals, suggesting that mammals and insects may have different selection encountered the varied plant-derived miRNAs from food. The other is the reads of xenomes from silkworm hemolymph were lower than those from mammals^12^. According to the current studies on miRNAs, the functions of miRNAs appear to be associated with their abundances^21^. Gavriel *et al.* reported only the most abundant miRNAs in a cell mediated significant target suppression^21^. Thus, there was a question whether miRNA with low abundance could play roles in physiological processes. Based on our data, the answer is no. Zhang *et al.* found rice miR168 regulated the level of cholesterol by targeting to LDLRAP1^12^. Zhou *et al.* reported honeysuckle miR2911 inhibited H1N1-PB2 and AS1 to suppress viral infection^20^. The abundances of the above two miRNAs (miR168 and miR2911) were very high in mice tissues after ingestion, which may provide the basis for the miRNAs playing roles in mice. Therefore, we can speculate that cross-kingdom miRNA transfer is a conserved phenomenon, however, not all plant-derived miRNAs have influence on the physiological progress of recipient animal organism. It is still unclear how plant-derived miRNAs resist to herbivore RNAses. The differential stability or resistance to RNAses in recipient animal tissues might be crucial for their functions. Further analysis will be required to clarify the underlying mechanism.

## Material and Methods

### Identification of mulberry-derived miRNAs in silkworm hemolymph

The hemolymph small RNA data of silkworm (GSE48168) was downloaded from NCBI (http://www.ncbi.nlm.nih.gov/). Mulberry-derived miRNAs identified from mulberry leaves small RNA library^18^ were aligned with silkworm hemolymph small RNA sequences.

### Preparations of the silkworm hemolymph and tissue samples

Silkworm larvae were fed mulberry leaves under a 12 h–light/12 h-dark cycle at 25 °C with relative humidity of 75%. Silkworm hemolymph was collected from fifth instar day-5 larvae using sterile tubes containing a few crystals of phenylthiourea. The silkworm larvae were dissected. The brain, prothoracic gland, salivary gland, gut, malpighian tubule, ovary, testis, fat body, and silk gland samples were collected and washed three times with cold 0.75% NaCl. Hemolymph samples were centrifuged at 3000 × g for 10 min at 4 °C. The supernatants of hemolymph, tissue samples were stored at −80 °C until use.

### Tissues from silkworms fed synthetic miR166b

300 pmol of synthetic miR166b (UCGGACCAGGCUUCAUUCCCC) with a 2’-O-methyl modified terminal nucleotide (RIBOBIO, China) was daubed on ~1 cm^2^ pieces of mulberry leaf. The synthetic miR166b was dissolved in RNase-free water. A single piece of leaf was fed to each silkworm larvae in the fifth instar day-5 after the miRNA liquid dried. The hemolymph, fat body, and silk gland were collected at 0.5 h, 3 h, 6 h and 12 h after the larvae ate the whole piece of leaf. Control (0 h) silkworms were only fed mulberry leaves. Each group contained five larvae.

### RNA extraction, T-A cloning and Sanger sequencing

Total RNA from the fat body and silk gland was extracted using RNAiso Plus (D9108A, Takara, China) according to the manufacturer’s instructions. The small RNA was purified using mirVana™ PARIS™ Kit (AM1556, Ambion, USA) and eluted in 25 μl non-nuclease water. Total RNA from fat body and silk gland and small RNA from hemolymph were reverse transcribed to cDNA in 10 μl reactions using M-MLV (D2641B, Takara, China) and specific reverse transcriptional primers for each miRNA. Stem-loop PCR was performed using miRNA specific forward and reverse primers (supplementary Table S5). The PCR products were cloned into pMD19-T vector (D102A, Takara, China). We randomly picked 60 monoclones for each miRNA, and of these, chose 50 clones that could be verified by bacterial PCR for Sanger sequencing with M13 primer.

### Measurement of miRNA copy number counts by droplet digital PCR

To standardize the amount of tissue at five time points, we reverse transcribed the same amount of total RNA from the silk gland and fat body, or small RNA from hemolymph at each time point. Total RNA (2.4 μg) from silkworm fat bodies or silk glands, or small RNA (800 ng) from hemolymph before or after oral synthetic miR166b at each of five time points was reverse transcribed in 15 μl reactions using the TaqMan microRNA reverse transcription kit (43366596, ABI, USA). ddPCR was performed using the QX100 droplet generator and droplet reader. Each PCR reaction was carried out in a 20 μl volume containing 10 μl 2X ddPCR supermix (Bio-rad, USA), 1.5 μl cDNA, 1 μl miR166b-specific hydrolysis probe assay (4427975, Life Technologies, USA), and 7.5 μl non-nuclease water. The 20 μl reaction was divided by the droplet generator and detected by the droplet readers according to the manufacturer’s instructions.

### Materials for RNA-seq

The 450 pmol synthetic miR166b (UCGGACCAGGCUUCAUUCCCC) with 2’-O-methyl modification on its terminal nucleotide (RIBOBIO, China) was daubed on a ~1 cm^2^ piece of mulberry leaf. Piece of leaf were fed to silkworm larvae in the fifth instar day-2 after the miRNA liquid dried. Whole silkworm larvae were collected at 3 h after ingestion of an entire piece leaf, and this experimental group (FED-miR166b) was stored at −80 °C until use. A control group of silkworm (FED-control) was only fed mulberry leaves. Each group contained three larvae. Total RNA from whole larvae were extracted by RNAiso Plus (D9108A, Takara, China) according to manufacturer’s instructions. Agilent 2100 was used to ensure the integrity of total RNA. cDNA libraries were constructed using the TruSeq RNA Sample Preparation v2 Guide (Illumina), and then sequenced by Illumina HiSeq^TM^ 2000. The obtained raw data from solexa sequencing were deposited on the Short Read Archive of NCBI (http://www.ncbi.nlm.nih.gov/sra/) with accession number SRP051555.

### Transcriptome analysis before and after silkworm larvae were fed synthetic miR166b

The quality of raw data in the FED-control and FED-miR166b transcriptome was determined by CLC Genomics Workbench 5.5 following the manufacturer’s instructions. After filtering the low quality, the clean reads were obtained that matched the silkworm genome and silkworm genes (http://www.silkdb.org/silkdb/doc/download.html) by SOAP2^22^,^23^. The distribution and coverage of clean reads on the silkworm genome and genes were analyzed using CLC Genomics Workbench 5.5. Expression analysis of each gene in two transcriptomes was performed as follows. Briefly, the expression level of each gene was normalized using the reads per kb per million reads (RPKM) method^24^. P value < 0.05 and false discovery rate (FDR) < 0.001 were used as thresholds for gene expression. Genes with absolute values of the log_2_ of the ratio of RPKMs (FED-miR166b/FED-control) that were more than 1 were regarded as a differentially expressed. Values of more than 1 indicated up-regulated genes, and values of less than −1 indicated down-regulated genes. The annotations of differentially expressed genes were performed by aligning against the non-redundant protein sequences (nr) database using BLASP (http://blast.st-va.ncbi.nlm.nih.gov/Blast.cgi?PROGRAM=blastp&PAGE_TYPE=BlastSearch&LINK_LOC=blasthome).

### Target site prediction for 30 differentially expressed genes and silkworm full-length cDNA for miR166b

Silkworm full-length cDNA sequences were downloaded from KAIKObase (http://sgp.dna.affrc.go.jp/FLcDNA/). Target sites prediction for 30 differentially expressed genes and silkworm full-length cDNA for miR166b was performed using Miranda software (http://www.microrna.org/microrna/getDownloads.do). The criteria were set as follows. (1) The “seed region” of miR166b (containing the 2^nd^ to 8^th^ nucleotides of the mature miRNA from 5’ to 3’) should match the target genes (30 differentially expressed genes) with perfect complementary. (2)The free energy of the hybrid should be less than −25 kcal/mol.

### RT-PCR detection of potential target genes

The 450 pmol synthetic miR166c (UCUCGGACCAGGCUUCAUUCC) and synthetic miR167e (UGAAGCUGCCAGCAUGAUCUG) with 2’-O-methyl modification on their terminal nucleotides (RIBOBIO, China) were fed to silkworm larvae as above described. Each group contained three larvae. The larvae in control group (FED-control) were only fed on mulberry leaves. Total RNA of FED-miR166b, FED-miR166c, FED-miR167e, and FED-control from whole larvae were extracted by RNAiso Plus (D9108A, Takara, China) according to manufacturer’s instructions. Total RNA were reverse transcribed to cDNA by using PrimeScript RT reagent Kit with gDNA Eraser (RR047A, Takara, China). The cDNA was then diluted four times and used as the template to perform the RT-PCR with gene specific forward and reverse primers, as listed in supplementary Table S6. The PCR reactions were performed in ABI Step One Plus (Applied Biosystems, USA) using SYBR Premix Ex TaqTM II (RR820A, TakaRa, China) as the following conditions: 95 °C for 30 s, 40 cycles of 95 °C for 5 s, and 60 °C for 30 s. The translation initiation factor 4 A was used as an inner control. All reactions were assayed in triplicated. The relative expression level of genes was calculated using 2^−∆∆Ct^ method.

### Phenotypic investigation of silkworm larvae fed on mulberry leaves with/without synthetic miR166b

The FED-miR166 and FED-control groups contained five sub-groups, separately, and each sub-group contained 10 larvae. The weight of larva, rate of wandering, and rate of survival in each groups were investigated. The weight of pupa and cocoon were measured at 10 days after ingestion of synthetic miR166b. Student’s t test (*p < 0.05) was performed to determine the statistical significance according to the graphpad software (http://www.graphpad.com/quickcalcs/ttest1.cfm).

## Additional Information

**How to cite this article**: Jia, L. *et al.* Nonfunctional ingestion of plant miRNAs in silkworm revealed by digital droplet PCR and transcriptome analysis. *Sci. Rep.* **5**, 12290; doi: 10.1038/srep12290 (2015).

## Supplementary Material

Supplementary Information

## Figures and Tables

**Table 1:** Eight mulberry miRNAs in silkworm hemolymph identified by solexa sequencing.

miRNA name	Sequence	The reads in the 1^st^ small RNA library	The reads in the 2^nd^ small RNA library
mno-miR166c**	UCUCGGACCAGGCUUCAUUCC	5	3
mno-miR166b**	UCGGACCAGGCUUCAUUCCCC	2	11
mno-miR167e	UGAAGCUGCCAGCAUGAUCUG	1	1
mno-miR396b	UUCCACAGCUUUCUUGAACUG	1	–
mno-miR159a	UUUGGAUUGAAGGGAGCUCUG	2	–
mno-miR162	UCGAUAAACCUCUGCAUCCAG	1	–
mno-miR156c	UUGACAGAAGAUAGAGAGCAC	–	2
mno-miR398	UGUGUUCUCAGGUCGCCCCUG	–	2

^**^miRNAs reported in the mulberry genome by He *et al.*^17^. “–” means the relative sequence was not detected in the hemolymph small RNA libraries.

**Table 2:** TA-cloning and Sanger sequencing results for mulberry miRNAs identified in silkworm hemolymph.

	1^st^ sequencing		2^nd^ sequencing	
Name	correct/Total clones	Rate	correct/Total clones	Rate
mno-miR169a	2/39	5.13%	0/37	0.00%
mno-miR166b	25/40	62.50%	26/39	66.67%
mno-miR166c	21/37	56.76%	32/46	69.56%
mno-miR167e	27/49	55.10%	15/46	32.61%
mno-miR396b	19/42	45.24%	16/32	50.00%
mno-miR159a	17/24	70.83%	–	–
mno-miR156c	14/39	35.90%	–	–
mno-miR398	20/29	68.97%	–	–
mno-miR162	0/49	0.00%	–	–
mno-miR535	0/38	0.00%	–	–
mno-miR168b	0/42	0.00%	–	–
mno-miR172a	0/39	0.00%	–	–

Results are shown for four control mulberry miRNA (mno-miR169a, mno-miR535, mno-miR168b, and mno-miR172a) and the eight mulberry miRNAs (mno-miR166b, mno-miR166c, mno-miR167e and mno-miR396b, mno-miR159a, mno-miR156c, mno-miR398, mno-miR162) in hemolymph. The TA-cloning of the five miRNAs (mno-miR169a, mno-miR166b, mno-miR166c, mno-miR167e, and mno-miR396b) was performed twice. Other miRNAs was performed once. We randomly selected 50 TA-clones for each miRNA for Sanger sequencing, but there were a number of double clones and failed sequencing reactions, so that the total clone number was less than 50. Clones were considered “correct” if the sequence of the clone was identical to the sequence of the corresponding miRNA. The “rate” indicates the sequencing frequency of correct clones relative to the total clones. “-” indicates that we did not clone the miRNA in the hemolymph.

**Table 3:** TA-cloning and Sanger sequencing results for mulberry miRNAs identified in silkworm tissues.

	Fat body (C/T)		Silk gland (C/T)								
miRNA name	1st sequencing	2nd sequencing	1st sequencing	2nd sequencing	Brain (C/T)	Prothoracic gland (C/T)	Salivary gland (C/T)	Gut (C/T)	Malpighian tubule (C/T)	Ovary (C/T)	Testis (C/T)
mno-miR169a	2.63%	0.00%	0.00%	0.00%	0.00%	2.17%	0.00%	0.00%	0.00%	2.33%	0.00%
mno-miR166b	20.55%	76.19%	56.76%	59.46%	53.66%	31.71%	9.09%	8.57%	10.81%	25.00%	13.33%
mno-miR166c	54.05%	95.45%	72.22%	93.02%	59.52%	61.76%	20.83%	24.39%	42.11%	31.82%	37.50%
mno-miR167e	12.50%	2.27%	13.51%	2.33%	25.64%	9.76%	6.67%	8.11%	20.00%	7.32%	8.16%
mno-miR396b	73.17%	65.96%	55.81%	45.45%	87.23%	66.67%	36.84%	31.71%	48.78%	34.09%	22.44%
mno-miR159a	0.00%	–	5.00%	–	–	–	–	–	–	–	–
mno-miR156c	8.33%	–	2.17%	–	–	–	–	–	–	–	–
mno-miR398	0.00%	–	0.00%	–	–	–	–	–	–	–	–
mno-miR162	0.00%	–	0.00%	–	–	–	–	–	–	–	–

Results are shown for one control mulberry miRNA (mno-miR169a) and four mulberry miRNAs (mno-miR166b, mno-miR166c, mno-miR167e and mno-miR396b) in silkworm tissues (brain, prothoracic gland, salivary gland, gut, malpighian tubule, ovary, testis, fat body, and silk gland). The TA-cloning of the five miRNAs (mno-miR169a, mno-miR166b, mno-miR166c, mno-miR167e, and mno-miR396b) was performed twice in fat body and silk gland, once in other tissues. The percentage represents the sequencing frequency of correct clones relative to the total clones. “–” indicates that we did not clone the miRNA in the fat body, silk gland, brain, prothoracic gland, salivary gland, gut, malpighian tubule, ovary, or testis.

**Table 4:** Genes that were differentially expressed before and after silkworms were fed synthetic miR166b.

Gene ID	FED-control	FED-166b	log_2_ratio	Up/Down-Regulated	E-value	Annotation (nr database)	Function	Species	Refences
Bm_scaffold779_2	2.595	0.924	−1.49	Down	0	heat shock protein 68	stress response	*H. zea*	Zhang and Denlinger, 2010^24^
Bm_nscaf2818_080	27.183	57.649	1.085	Up	1.00E-29	protease inhibitor-like protein	immunity	*A. mylitta*	Gandhe *et al.* 2006^25^
Bm_nscaf3098_40	8.832	52.509	2.572	Up	1.00E-126	gloverin 1 precursor	immunity	*B. mori*	Kawaoka *et al.* 2008^26^
Bm_nscaf3050_1	0.607	1.886	1.634	Up	0	glucose dehydrogenase [acceptor]-like, partial	immunity	*M. sexta*	Diana *et al.* 1994^27^
Bm_nscaf2556_12	25.945	65.987	1.347	Up	2.00E-149	attacin precursor	immunity	*B. mori H. cecropia*	Sugiyama *et al.* 1995^28^ Carlsson *et al.* 1991^29^
Bm_nscaf1071_24	26.328	61.537	1.225	Up	2.00E-32	cecropin-D precursor	immunity	*B. mori*	Yang *et al.* 1999^30^
Bm_nscaf2556_13	112.806	232.042	1.041	Up	2e-150	attacin precursor	immunity	*B. mori*	Sugiyama *et al.* 1995^28^ Carlsson *et al.* 1991^29^
Bm_nscaf2655_045	2.21	4.466	1.015	Up	1.00E-174	division abnormally delayed protein-like	immunity	*Drosophilia*	Zhu and Zhang, 2013^31^
Bm_nscaf2970_047	4.009	1.501	−1.417	Down	7.00E-130	mucin-17-like	immunity	*Trichoplusia ni*	Wang and Granados, 1997^32^
Bm_nscaf2814_10	2.787	1.096	−1.347	Down	0	serine protease inhibitor 19 precursor	immunity	*M. sexta*	An *et al.* 2011^33^
Bm_nscaf1071_25	6.426	18.529	1.528	Up	9.00E-31	antibacterial peptide enbocin 2 precursor	immunity	*B. mori*	Kim *et al.* 1998^34^ Kaneko *et al.* 2007^35^
Bm_nscaf2883_004	2.746	7.882	1.521	Up	4.00E-58	golgi-specific brefeldin A-resistance guanine nucleotide exchange factor 1-like	immunity	*a cell line*	Panda D, 2011^36^
Bm_nscaf2847_032	7.823	15.792	1.013	Up	1.00E-120	maltase 1-like	digestion	*A. gambiae A. aegypti*	Zheng *et al.* 1995^37^ James *et al.* 1989^38^
Bm_nscaf3032_113	0.859	2.096	1.287	Up	0	juvenile hormone esterase-like	hydrolyzing JH, transporting JH	*H. virescens*	Hanzlik *et al.* 1989^39^
Bm_nscaf2954_01	2.511	0.762	−1.72	Down	0	protein odd-skipped-like	development	*Drosophilia*	Gao *et al.* 2011^40^
Bm_nscaf3045_44	1.965	0.59	−1.735	Down	5.00E-73	c-Cbl-associated protein isoform A (CAP)	development	*B. mori*	Georgomanolis *et al.* 2009^41^
Bm_nscaf2865_098	1.047	0.309	−1.762	Down	0	LOW QUALITY PROTEIN: voltage-dependent T-type calcium channel subunit alpha-1G-like	development	*Drosophilia*	Dason *et al.* 2009^42^
Bm_nscaf2993_283	2.588	1.053	−1.298	Down	0	synaptotagmin I	development	*Drosophilia*	Yoshihara *et al.* 2002^43^
Bm_nscaf2886_33	0.974	2.736	1.49	Up	0	kinesin-like protein unc-104-like	development	*Drosophilia*	Kern *et al.* 2013^44^
Bm_nscaf2883_108	1.58	0.358	−2.14	Down	0	WD repeat-containing protein 67-like	cytoskeleton	*Saccharomyces cerevisiae*	Walter *et al.* 2003^45^
Bm_nscaf2795_061	66.485	21.505	−1.628	Down	0	low molecular mass 30 kDa lipoprotein 19G1-like	unknown	–	–
Bm_nscaf2795_060	453.713	146.829	−1.628	Down	0	low molecular 30 kDa lipoprotein PBMHP-12-like	unknown	–	–
Bm_nscaf2795_062	16.964	5.728	−1.566	Down	0	low molecular mass 30 kDa lipoprotein 21G1-like	unknown	–	–
Bm_nscaf2795_064	7.011	2.058	−1.769	Down	0	low molecular mass 30 kDa lipoprotein 19G1-like precursor	unknown	–	–
Bm_nscaf3003_019	2.588	9.563	1.885	Up	3.00E-75	actin cytoskeleton-regulatory complex protein PAN1-like	unknown	–	–
Bm_nscaf2903_06	2.987	6.29	1.075	Up	2.00E-158	uncharacterized protein LOC101737815	unknown	–	–
Bm_nscaf2529_069	1.619	0.663	−1.288	Down	0	uncharacterized protein LOC101738767	unknown	–	–
Bm_scaffold700_2	2.896	6.168	1.091	Up	6.00E-141	neuroligin-2-like	unknown	–	–
Bm_nscaf2575_112	0.001	0.714	9.479	Up	1.00E-159	Bardet-Biedl syndrome 1 protein-like	unknown	–	–
Bm_nscaf3063_090	3.78	12.138	1.683	Up	5.00E-25	myotubularin-related protein 3-like	unknown	–	–

Gene expression levels (reads per kb per million reads, RPKM) were determined prior to feeding (FED-control) or after feeding of synthetic miR166b (FED-miR166b). The “FED-control” and “FED-166b” represents FED-control-RPKM and FED-166b-RPKM, respectively. The “log_2_ratio” means log_2_ (FED-166b-RPKM/FED-control-RPKM). The “Up/down-regulated” means Up-Down-Regulation (FED_166b/FED_control). *H. zea*, *A.mylitta*, *B. mori*, *M. sexta*, *H. cecropia*, *A. gambiae*, *A. aegypti, H. virescens*, and *S. cerevisiae* represent *Helicoverpa zea*, *Antheraea mylitta*, *Bombyx mori*, *Manduca sexta*, *Hyalophora cecropia*, *Anopheles gambiae*, *Aedes aegypti*, *Heliothis virescens*, and *Saccharomyces cerevisiae*, respectively.
