# Supplementary material for: Nonfunctional ingestion of plant miRNAs in silkworm revealed by digital droplet PCR and transcriptome analysis
Source: Sci Rep. 2015 Jul 21;5:12290. doi: 10.1038/srep12290 (PMC4508662; doi:10.1038/srep12290)
Supplement: Supplementary Information [file srep12290-s1.pdf]

Nonfunctional ingestion of plant miRNAs in silkworm revealed by digital droplet PCR and transcriptome analysis

Ling Jia [1], Dayan Zhang [1], Zhonghuia Xiang [1], Ningjia He [1,*]

[1]State Key Laboratory of Silkworm Genome Biology, Southwest University, Beibei, Chongqing 400715, P. R. China

*Corresponding author: Ningjia He

Tel: +86-23-6825-0797

Fax: +86-23-6825-1128

Email addresses: hejia@swu.edu.cn

Supplementary Tables and Figures

Supplementary Table S1 TA-cloning and Sanger sequencing for mulberry miRNAs identified in silkworm tissues.

miRNA name	Fat body (C / T)		Silk gland (C / T)		Brain (C / T)	Prothoracic gland (C / T)	Salivary gland (C/ T)	Gut (C / T)	Malpighian tubule (C / T)	Ovary (C / T)	Testis C / T)
	1st sequencing	2nd sequencing	1st sequencing	2nd sequencing							
mno-miR169a	1/38	0/38	0/28	0/42	0/35	1/46	0/35	0/39	0/38	1/43	0/45
mno-miR166b	15/73	32/42	42/74	22/37	22/41	13/41	3/33	3/35	4/37	10/40	6/45
mno-miR166c	20/37	42/44	26/36	40/43	25/42	21/34	5/24	10/41	16/38	14/44	12/32
mno-miR167e	5/40	1/44	5/37	1/43	10/39	4/41	2/30	3/37	8/40	3/41	4/49
mno-miR396b	30/41	31/47	24/43	20/44	41/47	26/39	14/38	13/41	20/41	15/44	11/49
mno-miR159a	0/40	-	1/20	-	-	-	-	-	-	-	-
mno-miR156c	1/12	-	1/46	-	-	-	-	-	-	-	-
mno-miR398	0/49	-	0/48	-	-	-	-	-	-	-	-
mno-miR162	0/42	-	0/40	-	-	-	-	-	-	-	-

C / T means correct clones / Total clones. “-” indicates that we did not clone the miRNA in the fat body, silk gland, brain, prothoracic gland, salivary gland, gut, malpighian tubule, ovary, and testis.

Supplementary Table S2 The copy number counts of miR166b in silkworm hemolymph (Hem), fat body (FB), and silk gland (SL) before (0 h) and following ingesting synthetic miR166b (0.5 h, 3 h, 6 h, and 12 h).

**Sample**	**miR166(counts/μl)**		**Average value of miR166(counts/μl)**
	**1st well**	**2nd well**	
Hem-0 h	22.5	21.7	22.1
Hem-0.5 h	562	745	653.5
Hem-3 h	377	456	416.5
Hem-6 h	28.9	30.4	29.65
Hem-12 h	47.6	59.8	53.7
FB-0 h	358	381	369.5
FB-0.5 h	1160	1100	1130
FB-3 h	511	552	531.5
FB-6 h	206	217	211.5
FB-12 h	140	121	130.5
SL-0 h	192	212	202
SL-0.5 h	162	190	176
SL-3 h	114	84	99
SL-6 h	114	105	109.5
SL-12 h	109	108	108.5

Supplementary Table S3 The predicted information for miR166b target genes.

<table>
<tr><th>Potential target gene</th><th>Potential target gene annotation</th><th>Gene in derived tissues [1, *]</th><th>Score</th><th>Energy (kcal/mol)</th><th>Length (nt)</th><th>Position start</th><th>Position end</th><th>Match condition</th></tr>
<tr><td>fner47P07</td><td>PREDICTED: similar to GA15241-PA [Tribolium castaneum]</td><td>nerve system + brain, larva</td><td>170</td><td>-41.45</td><td>1258</td><td>305</td><td>327</td><td>Query: 3’ ccCCTT--ACTTCGGACCAGGCt 5’
|||| || :||||||||||
Ref: 5’ ggGGAATGTGTGGCCTGGTCCGa 3’</td></tr>
<tr><td>fwd-23F03</td><td>PREDICTED: similar to Cpsf6 protein, partial [Strongylocentrotus purpuratus]</td><td>wing disc</td><td>161</td><td>-30.66</td><td>1273</td><td>827</td><td>850</td><td>Query: 3’ ccCCTTAC--TTCG-GACCAGGCt 5’
|||||| |||: |||||||
Ref: 5’ ggGGAATGCAAAGTGATGGTCCGc 3’</td></tr>
<tr><td>fprW24O02</td><td>PREDICTED: probable phosphorylase b kinase regulatory subunit alpha-like isoform 2 [Bombus terrestris]</td><td>prothoracic gland, W-stage</td><td>164</td><td>-29.99</td><td>5840</td><td>2633</td><td>2653</td><td>Query: 3’ cccctTACTTCGGACCAGGCt 5’
:||::|:||||||||
Ref: 5’ aagctGTGGGGTCTGGTCCGg 3’</td></tr>
<tr><td>fmxg11G04</td><td>PREDICTED: calcium-binding mitochondrial carrier protein Aralar1-like isoform 1 [Nasonia vitripennis]</td><td>maxillary galea</td><td>172</td><td>-29.95</td><td>4344</td><td>1687</td><td>1708</td><td>Query: 3’ ccccTTACTT-CGGACCAGGCt 5’
|:|||| ||||||||||
Ref: 5’ ccaaAGTGAAGGCCTGGTCCGt 3’</td></tr>
<tr><td>fe8d35D03</td><td>GK17955 [Drosophila willistoni]</td><td>embryo day 8</td><td>157</td><td>-28.73</td><td>5755</td><td>644</td><td>664</td><td>Query: 3’ ccccTTACTTCGGACCAGGCt 5’
|: | ||||||||||
Ref: 5’ tgacAGGGCTGCCTGGTCCGg 3’</td></tr>
</table>

<table>
<tr><td>fwgP16B11</td><td>olfactory receptor [Bombyx mori]</td><td>wing, pupa day 2-8</td><td>158</td><td>-27.79</td><td>5590</td><td>2091</td><td>2112</td><td>Query: 3’ ccCCTTAC-TTCGGACCAGGCt 5’
|| ||| ::| :|||||||
Ref: 5’ ggGGTATGTGGGATTGGTCCGc 3’</td></tr>
<tr><td>fufe34A12</td><td>PREDICTED: PAP-associated domain-containing protein 5-like [Apis mellifera]</td><td>unfertilized egg</td><td>161</td><td>-27.44</td><td>4400</td><td>337</td><td>360</td><td>Query: 3’ ccCCT-TACTTC--GGACCAGGCt 5’
||| | |:|| |||||||||
Ref: 5’ acGGAGACGGAGCACCTGGTCCGc 3’</td></tr>
<tr><td>NM_001043963.1</td><td>Bombyx mori yellow-fb mRNA, complete cds.</td><td>ovary/embryo/fat body</td><td>157</td><td>-27.38</td><td>1516</td><td>996</td><td>1017</td><td>Query: 3’ ccccttaCTTCGG-ACCAGGCt 5’
|:|||| |||||||
Ref: 5’ gtgttggGGAGCCGTGGTCCGc 3’</td></tr>
<tr><td>fwgP08C14</td><td>hook protein [Aedes aegypti]</td><td>wing, pupa day 2-8</td><td>163</td><td>-27.08</td><td>5275</td><td>4181</td><td>4199</td><td>Query: 3’ cccCTTACTTCGGACCAGGCt 5’
||| |:||||||||||
Ref: 5’ tctGAA--AGGCCTGGTCCGg 3’</td></tr>
<tr><td>fdpe24B10</td><td>REDICTED: similar to AGAP010191-PA [Tribolium castaneum]</td><td>diapaused egg</td><td>155</td><td>-26.85</td><td>2500</td><td>508</td><td>528</td><td>Query: 3’ ccCCTTACTTCGGACCAGGCt 5’
|| || : |:|||||||
Ref: 5’ cgGGTCTGTGACTTGGTCCGa 3’</td></tr>
<tr><td>DQ311279</td><td>Bombyx mori double-stranded RNA-binding zinc finger protein JAZ mRNA, complete cds.</td><td>testis/head/integument/mid gut/ silk gland</td><td>160</td><td>-26.71</td><td>1593</td><td>446</td><td>466</td><td>Query: 3’ ccCCTTACTTC-GGACCAGGCt 5’
|||| ||:| ::|||||||
Ref: 5’ tcGGAA-GAGGATTTGGTCCGg 3’</td></tr>
</table>

[*] indicateds the genes from the derived tissues in the silkworm full-length cDNA libraries. Score and energy mean the parameters of miR166b with its target predicted by miranda software. Position start and position end indicate the positions of target gene matching to miR166b. Query and Ref mean the sequences of miR166b and their target genes.

Supplementary Table S4 The expression level of 72 potential target genes for miR166b before and after silkworm ingested synthetic miR166b.

GeneID	Gene Length	FED_control-Expression	FED_166b-Expression	FED_control-RPKM	FED_166b-RPKM	log2 Ratio(FED_166b/FED_control)	Up-Down-Regulation(FED_166b/FED_control)	P-value	FDR
**DQ311279_KBtr3102131**	1293	0	8	0.0010	0.2348	7.8750	Up	0.0042	0.0105
**fphe18H11_KBtr3008861**	630	0	4	0.0010	0.2409	7.9123	Up	0.0652	0.1116
**fe8d35D03_KBtr3011035**	2115	2	6	0.0365	0.1076	1.5601	Up	0.1883	0.2900
**NM_001046951.1_KBtr3103491**	1821	2	12	0.0424	0.2500	2.5601	Up	0.0081	0.0190
**fmxg11G04_KBtr3006016**	1812	2	6	0.0426	0.1256	1.5601	Up	0.1883	0.2959
**e10025H07_KBtr3008407**	1617	2	2	0.0477	0.0469	-0.0248	Down	0.9839	0.9968
**fdpe27A10_KBtr3005579**	801	1	0	0.0482	0.0010	-5.5907	Down	0.4914	0.6103
**NM_001134242.1_KBtr3104129**	1251	2	0	0.0617	0.0010	-5.9475	Down	0.2436	0.3473
**MFB-22J01_KBtr3006608**	1293	6	4	0.1791	0.1174	-0.6098	Down	0.5296	0.6371
**fwgP08A16_KBtr3004462**	2037	10	17	0.1895	0.3166	0.7407	Up	0.2006	0.3028
**NM_001135202.1_KBtr3104178**	1734	12	4	0.2671	0.0875	-1.6098	Down	0.0451	0.0868
**fufe05M06_KBtr3003435**	2742	22	27	0.3097	0.3736	0.2706	Up	0.5187	0.6340
**fwgP08C14_KBtr3004464**	4116	62	50	0.5815	0.4609	-0.3352	Down	0.2222	0.3290
**fcaL50E18_KBtr3007892**	1464	24	22	0.6328	0.5702	-0.1504	Down	0.7259	0.8101
**ftes09J21_KBtr3002070**	3774	67	48	0.6853	0.4826	-0.5060	Down	0.0629	0.1127
**ftes01O21_KBtr3001806**	3957	106	110	1.0340	1.0547	0.0286	Up	0.8852	0.9088
**fe8d26E10_KBtr3000564**	966	26	31	1.0390	1.2176	0.2289	Up	0.5551	0.6476
**AB383140_KBtr3100772**	738	20	10	1.0461	0.5141	-1.0248	Down	0.0636	0.1113

**ftes40N05_KBtr3003068**	138	4	0	1.1189	0.0010	-10.1278	Down	0.0599	0.1097
**fner48F12_KBtr3001725**	1995	63	59	1.2190	1.1221	-0.1195	Down	0.6483	0.7341
**fcaL46I03_KBtr3010924**	1296	41	45	1.2212	1.3174	0.1095	Up	0.7279	0.8007
**bmmt22O18_KBtr3009956**	885	29	22	1.2649	0.9432	-0.4234	Down	0.3016	0.4148
**fcaL26A22_KBtr3007341**	183	6	3	1.2656	0.6220	-1.0248	Down	0.3298	0.4455
**ffbm37M15_KBtr3000868**	3384	116	147	1.3232	1.6482	0.3168	Up	0.0768	0.1285
**fwgP16B11_KBtr3004585**	219	8	5	1.4101	0.8663	-0.7029	Down	0.4053	0.5201
**EU073423_KBtr3101484**	3834	128	221	1.5433	2.6191	0.7631	Up	0.0000	0.0000
**fufe39C19_KBtr3004101**	1521	67	80	1.6985	1.9934	0.2310	Up	0.3351	0.6312
**fprW23F16_KBtr3009502**	2085	95	127	1.7588	2.3111	0.3940	Up	0.0437	0.0885
**fprW24O02_KBtr3009532**	3675	178	185	1.8697	1.9100	0.0308	Up	0.8396	0.8979
**fmgV52F14_KBtr3001347**	1233	62	70	1.9410	2.1541	0.1502	Up	0.5525	0.6545
**fufe34A12_KBtr3003983**	1713	87	103	1.9605	2.2814	0.2187	Up	0.2989	0.4185
**fwd-23F03_KBtr3000242**	589	30	29	1.9661	1.8681	-0.0738	Down	0.8447	0.8789
**fphe27I11_KBtr3009051**	279	17	9	2.3520	1.2239	-0.9424	Down	0.1116	0.1829
**fner46B19_KBtr3001701**	1737	112	128	2.4890	2.7960	0.1678	Up	0.3698	0.4909
**fphe06C05_KBtr3008553**	1422	102	70	2.7689	1.8678	-0.5680	Down	0.0107	0.0236
**fprW16K18_KBtr3009416**	1698	125	118	2.8417	2.6367	-0.1080	Down	0.5601	0.6437
**fufe11D11_KBtr3003523**	5334	421	509	3.0467	3.6206	0.2490	Up	0.0087	0.0197
**fner44J23_KBtr3001686**	1515	125	97	3.1849	2.4293	-0.3907	Down	0.0449	0.0887
**fcaL15I24_KBtr3007105**	2349	199	246	3.2702	3.9735	0.2810	Up	0.0408	0.0849
**fphe05O06_KBtr3008545**	1458	160	176	4.2361	4.5801	0.1127	Up	0.4756	0.6004
**fwgP14B05_KBtr3004550**	855	105	110	4.7405	4.8814	0.0423	Up	0.8310	0.9013
**fdpe24B10_KBtr3005478**	987	132	134	5.1625	5.1512	-0.0032	Down	0.9853	0.9853
**fdpe20E17_KBtr3005343**	3027	485	467	6.1849	5.8536	-0.0794	Down	0.3959	0.5167
**fcaL11N10_KBtr3007021**	192	33	23	6.6346	4.5451	-0.5457	Down	0.1642	0.2634

**AB177622_KBtr3102492**	2160	408	406	7.2833	7.1238	-0.0319	Down	0.7520	0.8880
**fufe21M15_KBtr3003731**	258	52	41	7.7801	6.0296	-0.3677	Down	0.2230	0.3240
**e10013A10_KBtr3008211**	2379	523	628	8.4861	10.0158	0.2391	Up	0.0051	0.0122
**e10004B12_KBtr3008032**	1662	1010	1131	23.4580	25.8198	0.1384	Up	0.0267	0.0570
**fner47P07_KBtr3001722**	1032	1112	1235	41.5474	45.3551	0.1265	Up	0.0339	0.1887
**fphe05E06_KBtr3008533**	489	653	657	51.5473	50.9774	-0.0160	Down	0.8404	0.8864
**MFB-15M01_KBtr3006466**	1884	4735	4624	97.0153	93.1232	-0.0591	Down	0.0476	0.0895
**fphe22F14_KBtr3008946**	909	3795	3811	160.9781	158.8964	-0.0188	Down	0.5703	0.7876
**AB064522_KBtr3102562**	396	0	0	-	-	-	-	-	-
**AB196705_KBtr3101600**	738	0	0	-	-	-	-	-	-
**AB330437_KBtr3101259**	1251	0	0	-	-	-	-	-	-
**AB425069_KBtr3100742**	1734	0	0	-	-	-	-	-	-
**DQ358082_KBtr3101752**	1257	0	0	-	-	-	-	-	-
**DQ443256_KBtr3101964**	1821	0	0	-	-	-	-	-	-
**fphe15F13_KBtr3011069**	1293	0	0	-	-	-	-	-	-
**ftes24P18_KBtr3002516**	270	0	0	-	-	-	-	-	-
**ftes49O09_KBtr3003295**	2166	0	0	-	-	-	-	-	-
**GU322819_KBtr3100270**	909	0	0	-	-	-	-	-	-
**MFB-15M07_KBtr3006467**	396	0	0	-	-	-	-	-	-
**NM_001043418.1_KBtr3102696**	396	0	0	-	-	-	-	-	-
**NM_001043453.1_KBtr3102731**	2160	0	0	-	-	-	-	-	-
**NM_001043521.1_KBtr3102794**	738	0	0	-	-	-	-	-	-
**NM_001043963.1_KBtr3103046**	1257	0	0	-	-	-	-	-	-
**NM_001046730.1_KBtr3103271**	1293	0	0	-	-	-	-	-	-
**NM_001110342.1_KBtr3103872**	3834	0	0	-	-	-	-	-	-
**NM_001172724.1_KBtr3104383**	909	0	0	-	-	-	-	-	-

**fe8d22B10_KBtr3000534**	435	0	0	-	-	-	-	-	-
**ftes10A11_KBtr3002088**	4407	0	0	-	-	-	-	-	-

Gene expression levels (reads per kb per million reads, RPKM) were determined prior to feeding (FED-control) or after feeding of synthetic miR166b (FED-miR166b).

Supplementary Table S5 Primers for stem-loop PCR of miRNAs.

miRNA name	Sequence (5'-3')	Reverse transcriptive primer	Forword primer (5'-3')	Reverse primer(5'-3')
**mno-miR166c**	TCTCGGACCAGGCTTCATTCC	GTCGTATCCAGTGCAGGGTCCGAGGTATTCGCACTGGATACGACggaatg	GGCGTCTCGGACCAGGCT	CAGTGCAGGGTCCGAGGTAT
**mno-miR167e**	TGAAGCTGCCAGCATGATCTG	GTCGTATCCAGTGCAGGGTCCGAGGTATTCGCACTGGATACGACcagatc	TCGAGAGTGAAGCTGCCAGC	CAGTGCAGGGTCCGAGGTAT
**mno-miR156c**	TTGACAGAAGATAGAGAGCAC	GTCGTATCCAGTGCAGGGTCCGAGGTATTCGCACTGGATACGACgtgctc	GCCGAGGAGATTGACAGAAGATA	CAGTGCAGGGTCCGAGGTAT
**mno-miR398**	TGTGTTCTCAGGTCGCCCCTG	GTCGTATCCAGTGCAGGGTCCGAGGTATTCGCACTGGATACGACcagggg	CCGAGGAGATGTGTTCTCAGGT	CAGTGCAGGGTCCGAGGTAT
**mno-miR166b**	TCGGACCAGGCTTCATTCCCC	GTCGTATCCAGTGCAGGGTCCGAGGTATTCGCACTGGATACGACggggaa	CGAGTTCGGACCAGGCTTCA	CAGTGCAGGGTCCGAGGTAT
**mno-miR159a**	TTTGGATTGAAGGGAGCTCTG	GTCGTATCCAGTGCAGGGTCCGAGGTATTCGCACTGGATACGACcagagc	CGAGAGTTTGGATTGAAGGGA	CAGTGCAGGGTCCGAGGTAT
**mno-miR162**	TCGATAAACCTCTGCATCCAG	GTCGTATCCAGTGCAGGGTCCGAGGTATTCGCACTGGATACGACctggat	CGAGGAGATCGATAAACCTCTGC	CAGTGCAGGGTCCGAGGTAT
**mno-miR396b**	TTCCACAGCTTTCTTGAACTG	GTCGTATCCAGTGCAGGGTCCGAGGTATTCGCACTGGATACGACcagttc	GCAGCAGTTCCACAGCTTTCTT	CAGTGCAGGGTCCGAGGTAT
**mno-miR535**	TGACAACGAGAGAGAGCACGC	GTCGTATCCAGTGCAGGGTCCGAGGTATTCGCACTGGATACGACgcgtgc	GCGAGTGTGACAACGAGAGAGA	CAGTGCAGGGTCCGAGGTAT
**mno-miR172a**	AGAATCTTGATGATGCTGCAT	GTCGTATCCAGTGCAGGGTCCGAGGTATTCGCACTGGATACGACatgcag	GCACGCAGAGAATCTTGATGATG	CAGTGCAGGGTCCGAGGTAT
**mno-miR168b**	TCGCTTGGTGCAGGTCGGGAA	GTCGTATCCAGTGCAGGGTCCGAGGTATTCGCACTGGATACGACttcccg	CGAGTTCGCTTGGTGCAGGT	CAGTGCAGGGTCCGAGGTAT
**mno-miR169a**	CAGCCAAGGATGACTTGCCGG	GTCGTATCCAGTGCAGGGTCCGAGGTATTCGCACTGGATACGACccggca	CAGGAGACAGCCAAGGATGACT	CAGTGCAGGGTCCGAGGTAT

Supplementary Table S6 Primers for RT-PCR of miR166b potential target genes and immunity-relatively genes.

Gene name	Forward Primer (5' to 3')	Reversed Primer (5' to 3')
**fwgP08C14**	TACACCGATAAAAGAGAACCCGA	CGTGTCTTGCTTCATTTCTATCTTG
**fwgP16B11**	ATTCCAGTGCCCATAATCGTCA	AGGATGCTAAAATATCCATGGATCC
**fe8d35D03**	GGCTCTGAACCACCCCACG	GCCCACAGACTATCCAATAATCG
**fmxg11G04**	GGGCGGTTCTCAAGTGGTGT	GGAAGTATATGGCGCTGAAGGGC
**fufe34A12**	TGGCGAATACCTAATTATGCGT	CAGTACGGAATGAACCGAACAC
**fdpe24B10**	CTATCCAGAGAAGAGGCAGAAGAA	GACCACATCGCTTTCTCTTCTTG
**NM_001043963.1**	TGTCGACTCACGAGTACGCCA	CTCCTCGACGAGCGGTTTCT
**fwd-23F03**	CACCTGGGCTGAGACCACCTA	TCCTGGCGGACCATCACTTT
**fprW24O02**	ACGGTAAGGTGGCGACAGTG	AATCAGTAACGGCTTTAGCGAGA
**fner47P07**	GGGATAAAATGCAGCGTCGAGTAGT	TGGCGGTGCTTGACCCTTTG
**DQ311279**	ATCCACCAAGACCCCCTCC	AGGAATGTTTTTCAAACTGTCCTTT
**Bm_nscaf1071_24**	TGTTCGCTATTGTTTTCGCC	CTTTTGCTTTTGCCAAGGTGT
**Bm_nscaf2556_13**	TACTCGGCCGGGGGTAAG	ACATTGGGCTCCCACGAAGAT
**Bm_nscaf2814_10**	GTGTGACCAACAACTGTGAGCCC	TACGGAAAATGCGAATGGACGA
**Bm_nscaf2818_080**	TATTGTATTGTTGTTTGTTGTGGCA	GGTCGCAGAAGCACTGTGGAAC
**Bm_nscaf3050_1**	GCAGGTGCTATCAACTCTCCTCAAT	TAAAGGTAATGGAGATTTCAAAGGA
**Bm_nscaf2970_047**	CTATGGTAGCGTCCAAGGAGAGA	CTCTTTCGAACTCCTCACGTAGC
**Translation initiation factor 4A**	TTCGTACTGGCTCTTCTCGT	CAAAGTTGATAGCAATTCCCT

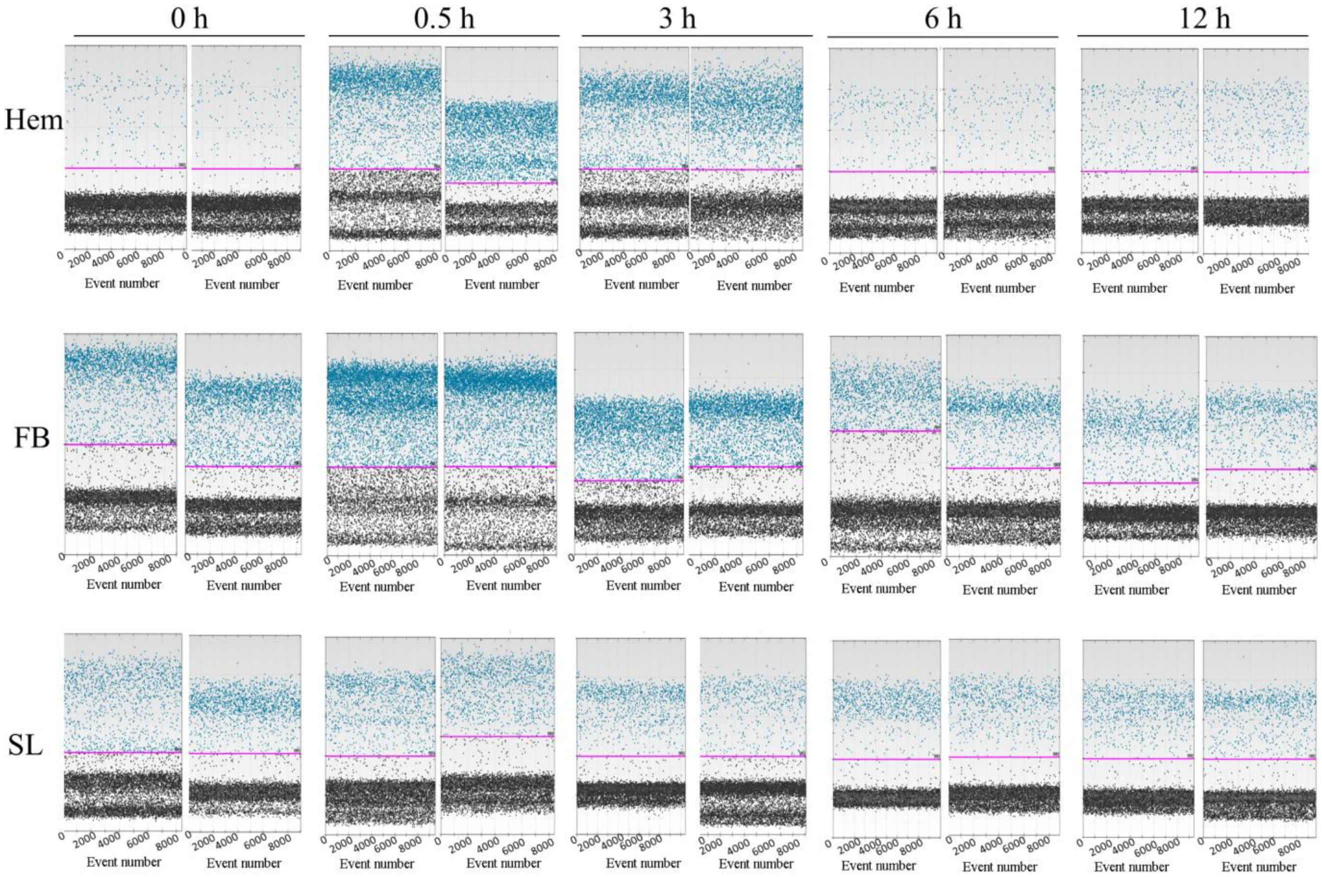

Supplementary Figure S1 Droplet digital PCR indicates the synthetic miR166b entered into the silkworm hemolymph, fat body, but not into silk gland after application of oral synthetic miR166b. The Y axis represents the signal intensity. The X axis represents the droplet numbers for a 20 μl PCR reaction divided by QX100 droplet generator. Hem: Hemolymph, FB: fat body, SL: silk gland. The purple line below represents the signals of NTC (no template control).

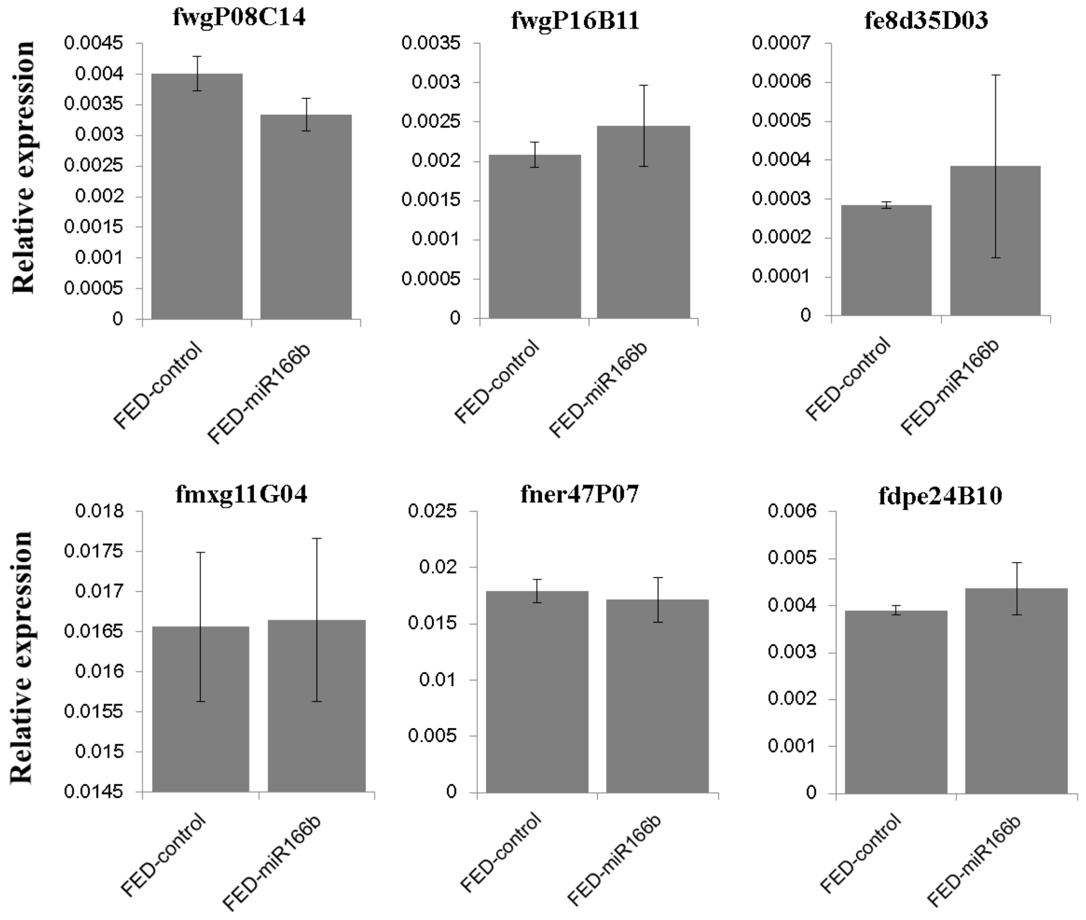

Supplementary Figure S2 The expression levels of 6 potential miR166b target genes in fat body before and after silkworm larvae ingested synthetic miR166b. FED-control represents the silkworm larvae were only fed on mulberry leaves. FED-miR166b means the silkworm larvae were fed on a piece of mulberry leaf containing synthetic miR166b. Statistical significance was determined by Student’s t test (*, $p < 0.05$).

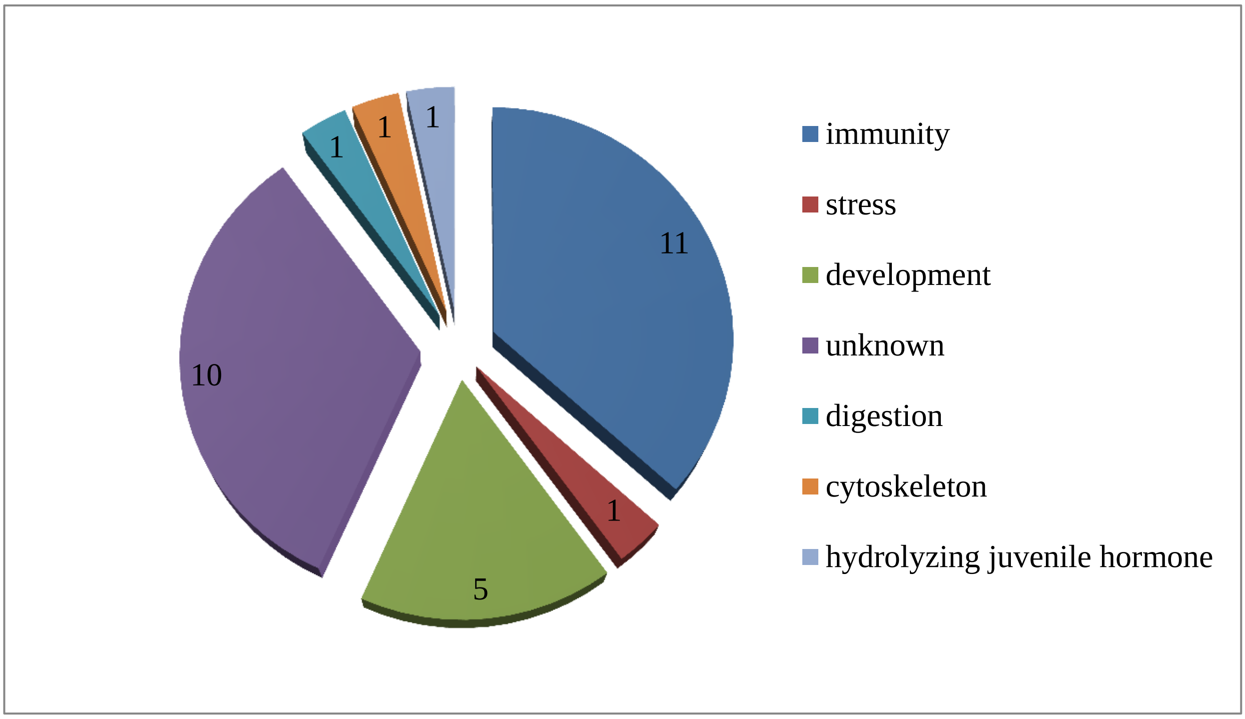

Supplementary Figure S3 Categorization of 30 differentially expression genes before and after ingestion synthetic miR166b by silkworm. The values indicate the gene numbers in each category.

References

1. Suetsugu ,Y. et al. Large scale full-length cDNA sequencing reveals a unique genomic landscape in a lepidopteran model insect, Bombyx mori. *G3 (Bethesda)* **3**, 1481-1492 (2013).
